# Global Health research abstracts: May ‘23

**DOI:** 10.1016/j.afjem.2023.05.005

**Published:** 2023-06-06

**Authors:** Dr. Jonathan Kajjimu

**Affiliations:** Faculty of Medicine, Mbarara University of Science and Technology, Mbarara, Uganda

## Abstract

The African Journal of Emergency Medicine, in partnership with several other regional emergency medicine journals, publishes abstracts from each respective journal. Abstracts are not necessarily linked to open access papers however, all abstracts are accessible without subscription.

**Annals of Emergency Medicine (North America)**

**Comparison of YEARS and adjust-unlikely D-dimer testing for pulmonary embolism in the emergency department.** de Wit, K., Al-Haimus, F., Hu, Y., Ikesaka, R., Chan, N., Ibrahim, Q., ... & Germini, F. (2022). *Annals of Emergency Medicine*.

DOI: https://doi.org/10.1016/j.annemergmed.2022.09.014

**Study objective**

We prospectively assessed the diagnostic accuracy of YEARS and a modified age-adjusted clinical decision rule (“Adjust-Unlikely”) for pulmonary embolism (PE) testing in the emergency department.

**Methods**

This study was conducted in tertiary care Canadian emergency departments. When the D-dimer was <500 ng/ml, PE was excluded. Pulmonary imaging for PE was performed when the D-dimer was ≥500 ng/ml. Patients were followed for 30 days, and PE outcomes were independently adjudicated. Physicians systematically recorded the presence or absence of YEARS items (PE most likely, hemoptysis, signs of deep venous thrombosis) prior to D-dimer testing and imaging. We analyzed the diagnostic accuracy of YEARS and the “Adjust-Unlikely” rule. Age adjustment (age x 10 in those >50 years old) was applied in patients where PE was not the most likely diagnosis and 500 ng/ml threshold when PE was most likely.

**Results**

One thousand seven hundred three patients were included, median age 62 (50, 74), 58% female, PE prevalence 8.0%. YEARS sensitivity for PE diagnosis was 92.6% (87.0, 96.0%) and specificity 45.0% (42.5, 47.5%). Adjust-Unlikely sensitivity was 100.0% (97.2, 100.0%) and specificity 32.4% (30.1, 34.8%). Posttest probability of PE in the group of patients with PE excluded by D-dimer between 500 ng/ml and the adjusted limit was 2.8% (1.6, 5.1%) for YEARS and 0.0% (0.0, 2.6%) for the “Adjust-Unlikely” rule.

**Conclusion**

The “Adjust-Unlikely” rule would modestly reduce imaging and identify all cases of PE. YEARS would substantially reduce imaging but miss 1 in 14 cases of PE.

Reproduced with permission.

**Canadian Journal of Emergency Medicine (North America)**

**Diagnosis and management of patients who present with narrow complex tachycardia in the emergency department.**

Linton, J. J., Eagles, C., Green, M. S., Nemnom, M. J., & Stiell, I. G. (2023). *Canadian Journal of Emergency Medicine*, 1–11.

DOI: https://doi.org/10.1007/s43678-023-00462-w

**Introduction**

While narrow complex tachycardia (NCT) is a common presentation to the emergency department (ED), little is known about its incidence in the ED or about emergency physician expertise in its diagnosis and management. We sought to compare cases of NCT due to primary arrhythmias to those with a rapid heart rate secondary to a medical issue, as well as to determine the accuracy of ED physician diagnosis and appropriateness of treatment.

**Methods**

We conducted a health records review at a large academic hospital ED staffed by 95 physicians and included consecutive adult patients over 7 months (2020–2021) with NCT (heart rate ≥ 130 bpm and QRS < 120 ms). Cases were reviewed for accuracy of ECG diagnosis and for correctness of treatment as per guidelines by an adjudication committee.

**Results**

We identified 310 ED visits (0.8% of all ED visits), mean age 65.1 years, 52.6% female. Primary arrhythmias accounted for 54.8%. ED physicians correctly interpreted 86.6% of ECGs. The most common arrhythmias and accuracy of ED physician ECG interpretation were atrial fibrillation 44.5% (95.1%), sinus tachycardia 24.2% (90.5%), atrial flutter 15.8% (61.5%), and supraventricular tachycardia (SVT) 12.9% (81.6%). Treatments were judged optimal in 96.5% of primary NCT and 99.3% in secondary NCT. Treatments were suboptimal for failure to reduce heart rate < 100 bpm prior to discharge in 2.1% of primary cases and failure to treat underlying cause in 0.7% of secondary cases.

**Conclusion**

NCT was found in 0.8% of all ED visits, with more being primary NCT. ED physicians correctly interpreted 86.6% of ECGs but had difficulty differentiating atrial flutter and SVT. They implemented appropriate care in most cases but sometimes failed to adequately control heart rate or to treat the underlying condition, suggesting opportunities to improve care of NCT in the ED.

Reproduced with permission.

**Emergencias (Europe)**

**External validation of the Glasgow Coma Scale-Pupils in patients with severe head injury.**

Barea-Mendoza, J. A., Llompart-Pou, J. A., Pérez-Bárcena, J., Quintana-Díaz, M., Serviá-Goixart, L., Guerrero-López, F., ... & Chico-Fernández, M. (2023). *Emergencias: Revista de la Sociedad Espanola de Medicina de Emergencias*, *35*(1), 39–43.

https://bit.ly/413ocny

**Objectives**

To compare the ability of the Glasgow Coma Scale (GCS) score, the GCS Pupils (GCS-P) score, and the Pupil Reactivity Score (PRS) to predict mortality in patients with severe head injury.

**Material and methods**

Retrospective analysis of all patients with severe head injury and initial GCS scores of 8 or lower on initial evaluation for whom records included pupil dilation information and clinical course after admission to intensive care units of participating hospitals. We assessed the ability of each of the 3 scores (GCS, GCS-P, and PRS) to predict mortality using discrimination analysis. Discrimination was estimated by calculating the areas under the receiver operating characteristic curves (AUC) and 95% CIs.

**Results**

A total of 1551 patients with severe head injury and pupil dilation records were studied. The mean age was 50 years, 1190 (76.7%) were males, and 592 (38.2%) died. No pupil dilation was observed in 905 patients (58.3%), 362 (23.3%) had unilateral mydriasis, and 284 (18.3%) had bilateral mydriasis. The GCS-P score was significantly better at predicting mortality, with an AUC of 0.77 (95% CI, 0.74-0.79), versus 0.69 (95% CI, 0.67-0.72) for the GCS, and 0.75 (95% CI, 0.72-0.77) for the PRS. As the GCS-P score decreased, mortality increased.

**Conclusion**

The GCS-P was more useful than the GCS for predicting death after severe head injury.

Reproduced with permission.

**Emergency Medicine Journal (Europe)**

**Rates of perceived medical errors and its correlation with work-related factors and personal distress among emergency physicians in China: a national cross-sectional study.**

Yan, S., Wang, J., Yin, X., Lv, C., Wu, J., Jiang, N., ... & Gong, Y. (2023). *Emergency Medicine Journal*, *40*(5), 320–325.

DOI: http://dx.doi.org/10.1136/emermed-2021-212041

**Purpose**

Medical errors are a global concern, and specifically, EDs are at considerable risk for medical errors. Few studies focus on the healthcare provider's self-perceived medical errors in hospitals, let alone the ED. Hence, this study explored perceived medical errors and their correlation with work-related factors and personal distress among physicians in EDs in China.

**Methods**

From July 2018 to August 2018, a national web-based cross-sectional study was conducted. The link to the web-based questionnaire was posted on the emergency physicians’ working platform, inviting Chinese licensed emergency physicians to participate anonymously in this survey. Our outcome of interest, medical errors, was investigated using self-reporting methods. Occupational stress was assessed using the Chinese version of the Effort-Reward Imbalance scale. The Patient Health Questionnaire, the subscale of the 10-item Positive and Negative Affect Schedule, the subscale of the validated Leiden Quality of Work Questionnaire and the 10-item Generalised Self-efficacy Scale were used to assess personal distress. Logistic regression analysis was used to determine factors significantly associated with perceived medical errors.

**Results**

A sample of 10,457 emergency physicians completed the survey. Almost half (43.63%) of physicians reported self-perceived medical errors during the previous 3 months. The rate of workplace verbal aggression, effort-reward imbalance and depressive symptoms were 81.81%, 78.39% and 35.71%, respectively. Medical errors were more likely to be reported among chief physicians, and those who reported the department was short-staffed for physicians, and who experienced workplace verbal aggression and intense work stress. Medical errors were significantly associated with negative affect and lower self-efficacy.

**Conclusion**

Self-perceived medical errors are prevalent among physicians working in EDs and are associated with their workplace environment and personal distress. Targeted interventions are required to reduce physicians’ workload and improve their working environment. Accounting for healthcare providers’ distress is imperative for reducing the incidence of medical errors and improving their health.

Reproduced with permission.

**Hong Kong Journal of Emergency Medicine (Asia)**

**Adverse prognostic factors for rescuing patients with acute myocardial infarction–induced cardiac arrest receiving percutaneous coronary intervention under extracorporeal membrane oxygenation.**

Ye, J. (2021). *Hong Kong Journal of Emergency Medicine*, 1024907921997614.

DOI: https://doi.org/10.1177/1024907921997614

**Background**

Acute myocardial infarction–induced cardiac arrest has high mortality rate.

**Objective**

To investigate the risk factors of extracorporeal membrane oxygenation combined with percutaneous coronary intervention in rescuing acute myocardial infarction–induced cardiac arrest.

**Methods**

Forty-three eligible patients were assigned into death and survival groups. Their general clinical data, treatment outcomes, and various indicators 24, 48, and 72 h after extracorporeal membrane oxygenation implantation were compared. The factors affecting clinical outcomes were determined by multivariate logistic regression analysis. A nomogram prediction model was constructed and validated.

**Results**

After removing extracorporeal membrane oxygenation device, 19 patients recovered and 24 died (mortality rate: 55.81%). The two groups had different conventional cardiopulmonary resuscitation duration, number of diseased vessels, distribution of culprit vessel, time from cardiac arrest to extracorporeal membrane oxygenation implantation, length of stay in critical care unit, and mean arterial pressure 24 and 48 h after extracorporeal membrane oxygenation implantation (*p* < 0.05). Left anterior descending as the culprit vessel, number of diseased vessels, conventional cardiopulmonary resuscitation duration, time from cardiac arrest to extracorporeal membrane oxygenation implantation, and mean arterial pressure 48 h after extracorporeal membrane oxygenation resuscitation were independent risk factors for death. The predicted mortality rate was 72.6%, and the actual concordance index (C-index) was 0.869. Such indices after internal and external validations were 0.861 and 0.848, respectively, suggesting a good concordance.

**Conclusion**

Left anterior descending as the culprit vessel, number of diseased vessels, conventional cardiopulmonary resuscitation duration, time from cardiac arrest to extracorporeal membrane oxygenation implantation, and mean arterial pressure 48 h after extracorporeal membrane oxygenation resuscitation are independent risk factors for patients with acute myocardial infarction–induced cardiac arrest undergoing extracorporeal membrane oxygenation combined with percutaneous coronary intervention.

Reproduced with permission.

